# Erratum: Prolonged monitoring of cerebral blood flow and autoregulation with diffuse correlation spectroscopy in neurocritical care patients

**DOI:** 10.1117/1.NPh.7.1.019801

**Published:** 2020-02-20

**Authors:** Juliette Selb, Kuan-Cheng Wu, Jason Sutin, Pei-Yi (Ivy) Lin, Parisa Farzam, Sophia Bechek, Apeksha Shenoy, Aman B. Patel, David A. Boas, Maria Angela Franceschini, Eric S. Rosenthal

**Affiliations:** a Massachusetts General Hospital, Optics at Martinos, Athinoula A. Martinos Center for Biomedical Imaging, Department of Radiology, Charlestown, Massachusetts, United States; b Massachusetts General Hospital, Department of Neurology, Boston, Massachusetts, United States

## Abstract

Corrected disclosures for the article “Prolonged monitoring of cerebral blood flow and autoregulation with diffuse correlation spectroscopy in neurocritical care patients.”

This article [*Neurophotonics* **5**, 045005 (2018) doi: 10.1117/1.NPh.5.4.045005] was originally published online on 13 November 2018 with an error in the Disclosures section, where information regarding potential conflicts of interest was reported incompletely.

Original:

The authors hold patents on the DCS technology. MAF has a financial interest in 149 Medical, Inc., a company developing DCS technology for assessing and monitoring cerebral blood flow in newborn infants. MAF’s interests were reviewed and are managed by Massachusetts General Hospital and Partners HealthCare in accordance with their conflict of interest policies.

Corrected:

MAF and DAB have financial interest in 149 Medical, Inc., a company developing DCS technology for assessing and monitoring cerebral blood flow in newborn infants, and in Dynometrics, Inc., a company that makes devices that use NIRS technology for athletes to evaluate muscle performance. MAF and DAB interests were reviewed and are managed by Massachusetts General Hospital and Partners HealthCare in accordance with their conflict of interest policies.

This article was corrected online on 11 February 2020.

